# Physiological, Transcriptomic, and Metabolomic Responses of *Brachiaria decumbens* Roots During Symbiosis Establishment with *Piriformospora indica*

**DOI:** 10.3390/biology15030215

**Published:** 2026-01-23

**Authors:** Man Liu, Xinyong Li, Wenke Zhang, Xinghua Zhao, Yuehua Sun, An Hu, Rui Zhang, Kai Luo

**Affiliations:** 1 School of Tropical Agriculture and Forestry, Hainan University, Haikou 570228, China; manliu0506@163.com (M.L.); zhangwenke0304@163.com (W.Z.); 19862143810@163.com (X.Z.); 17886749426@163.com (Y.S.); zhangrui@hainanu.edu.cn (R.Z.); 2 Tropical Crops Genetic Resources Institute, Chinese Academy of Tropical Agricultural Sciences, Haikou 571101, China; lixy051985@163.com (X.L.); huan@catas.cn (A.H.)

**Keywords:** *Brachiaria decumbens*, root endophyte, symbiosis, transcriptome, metabolome, multi-omics

## Abstract

*Brachiaria decumbens* is a high-yielding forage grass of major economic value in tropical regions. The root endophytic fungus *Piriformospora indica* is widely recognized for promoting plant growth and stress tolerance, yet its effects on *B. decumbens* remain poorly characterized. Here, we profiled root responses to *P. indica* colonization at 10 days after inoculation (dais; early stage) and 20 dais (late stage) during symbiosis establishment. Colonization was confirmed by phenotypic and physiological assessments, with inoculated plants showing enhanced root growth; colonized roots exhibited higher activities of catalase (CAT), superoxide dismutase (SOD), and peroxidase (POD), along with increased indole-3-acetic acid (IAA) levels, whereas malondialdehyde (MDA), jasmonic acid (JA), and the ethylene precursor 1-aminocyclopropane-1-carboxylic acid (ACC) were reduced. Transcriptome and metabolomic profiling identified 1884 and 1077 differentially expressed genes (DEGs) and 2098 and 1509 differentially accumulated metabolites (DAMs) at 10 dais (Pi10d vs. CK10d) and 20 dais (Pi20d vs. CK20d), respectively, and 3355 DEGs and 2314 DAMs between stages (Pi20d vs. Pi10d). Functional enrichment highlighted key pathways related to secondary metabolism, carbohydrate metabolism, and lipid biosynthesis. Differentially expressed transcription factors spanned multiple families, including MYB, AP2/ERF, MADS-box, and bZIP, consistent with broad transcriptional reprogramming during symbiosis establishment. Integrative multi-omics analysis further highlighted phenylpropanoid biosynthesis and α-linolenic acid metabolism as consistently co-enriched pathways, suggesting coordinated shifts in gene expression and metabolite accumulation across colonization stages. Collectively, these results provide a multi-layered resource and a framework for mechanistic dissection of the *P. indica*–*B. decumbens* interaction.

## Simple Summary

*Brachiaria decumbens* is an economically important forage grass widely cultivated in tropical regions. Although the root endophytic fungus *Piriformospora indica* is well known for enhancing plant growth and stress tolerance in many species, its effects on *B. decumbens* have not been well characterized. This study investigated the potential benefits of *P. indica* for *B. decumbens* and the underlying mechanisms involved. Colonized roots exhibited reduced stress signatures and altered levels of endogenous growth-related compounds. By integrating transcriptomic and metabolomic profiling across multiple colonization stages, we found that *P. indica* triggers extensive reprogramming of root biological processes. Several candidate regulatory proteins and metabolites also appeared to coordinate these responses. This study provides the first comprehensive, system-level view of the *P. indica*–*B. decumbens* interaction, offering a valuable foundation for improving the growth and stress resistance of this important forage to enhance feed quality and promote more sustainable livestock production.

## 1. Introduction

Beneficial soil microorganisms, including root-endophytic fungi, can improve crop performance by influencing plant nutrition, development, and stress responses [1,2]. *Piriformospora indica* (lately referred to as *Serendipita indica*) is an axenically cultivable, root-colonizing endophytic fungus originally isolated from the rhizosphere of a woody shrub in the Thar Desert, India [3]. In contrast to many obligate mycorrhizal fungi, *P. indica* can be maintained readily in vitro, which has facilitated both mechanistic studies and exploratory agricultural applications [4]. The fungus colonizes a broad range of hosts, including both monocots and dicots, and retains compatibility under diverse environmental conditions [5]. During colonization, septate hyphae enter the root cortex and form characteristic intracellular hyphal coils within cortical cells [6]. As a symbiont, fungal proliferation depends on host-derived carbon, linking successful colonization to host carbon status and potentially shaping outcomes under stress [7,8]. Consistent with this, numerous studies report that *P. indica* inoculation is associated with enhanced root development, improved nutrient acquisition, and increased tolerance to abiotic and biotic stresses across multiple plant systems [8,9,10,11,12]. Owing to its broad host range and axenic growth, *P. indica* has become a unique model for dissecting physiological and molecular mechanisms of beneficial plant–microbe interactions [11,13,14,15].

In recent years, plant responses to *P. indica* colonization have been characterized at physiological, transcriptional, and proteomic levels, providing insights into how beneficial endophytes modulate host growth and stress-related pathways [16]. For example, Jacobs et al. [17] showed that *P. indica* dampens innate immune responses in *Arabidopsis* roots by interfering with microbe-associated molecular pattern (MAMP)-triggered defenses, and that this effect is attenuated in jasmonate (JA) mutants. Zuccaro et al. [18] proposed a biphasic colonization program, with an initial biotrophic phase followed by a cell-death-associated phase, and identified candidate small secreted proteins harboring novel motifs. In tomato, Ntana et al. [11] reported metabolomic shifts linked to lignin-derived compounds and polyacetylenes during colonization. In Chinese cabbage, Hua et al. [19] found that growth promotion coincided with altered root metabolite profiles, including changes in auxin-related compounds, γ-aminobutyric acid (GABA), and polyunsaturated fatty acids. Moreover, stage-dependent transcriptional responses have been observed during symbiosis establishment, consistent with dynamic host reprogramming across early versus later colonization phases [20,21,22]. Together, these studies indicate that *P. indica* employs effective colonization strategies, while the magnitude and direction of host physiological and molecular responses can differ across plant species.

*Brachiaria decumbens* (Stapf) R.D. Webster (also known as signal grass) is a decumbent perennial C4 grass and a major tropical forage species, native to tropical Africa [23,24]. It develops a robust root system and is valued for broad soil adaptability, drought tolerance, and relatively low management requirements [25,26,27,28]. *B. decumbens* also provides favorable forage quality, with a reported dry matter digestibility of 35.6%, crude protein content of 8.3%, and average annual dry matter yields of 25.5 t ha^−1^ [23,29]. Accordingly, it is widely cultivated as pasture and forage across tropical and subtropical regions [28,30]. Extending the study of *P. indica* symbiosis beyond model and horticultural plants, *B. decumbens* represents a distinct physiological and ecological niche. As a tropical C4 perennial forage grass, it exhibits traits such as high light-use efficiency and adaptation to nutrient-poor soils, making it an agronomically critical and ecologically relevant system for investigating endophyte-mediated growth and stress resilience. However, the holistic mechanism by which *P. indica* promotes growth and stress resistance in *B. decumbens* remains to be established.

In this study, we investigated whether and how *P. indica* promotes growth in *B. decumbens*, and we explored associated physiological and molecular signatures during symbiosis establishment. Specifically, we quantified antioxidant enzyme activities (SOD, POD, and CAT), lipid peroxidation (malondialdehyde, MDA), and phytohormone-related metabolites, including jasmonic acid (JA), indole-3-acetic acid (IAA), and the ethylene precursor 1-aminocyclopropane-1-carboxylic acid (ACC), in roots of colonized plants and mock-inoculated controls. In parallel, we performed integrated transcriptomic and metabolomic analyses to characterize stage-dependent reprogramming and to identify pathways consistently associated with colonization. To our knowledge, this study provides the first integrated physiological and multi-omics characterization of the *P. indica*–*B. decumbens* interaction, delivering a system-level view into the holistic mechanism of growth promotion and stress resistance.

## 2. Materials and Methods

### 2.1. Biological Material, Inoculation, and Sampling

*Brachiaria decumbens* var. Reyan No. 3 seedlings, a cultivar with high genetic stability, were used in this study. Experiments were conducted in 2025 at the experimental station of Hainan University. Two-node vegetative cuttings were obtained from the Tropical Forage Germplasm Repository, Chinese Academy of Tropical Agricultural Sciences. After rinsing the roots with tap water, cuttings were transplanted into 17 cm diameter pots (two plants per pot). Pots were filled with a sterilized (121 °C, 3 consecutive days) 1:1 (*v*/*v*) mixture of potting soil and vermiculite. Fourteen days after transplanting, 60 pots (each containing two plants), totaling 120 uniform and healthy seedlings that were free from visible disease symptoms (e.g., leaf yellowing or spots), were selected for *P. indica* colonization assays.

The fungal strain *Piriformospora indica* DSM11827 was maintained on potato dextrose agar (PDA) and propagated at 28 °C for 7–10 days. Agar plugs (2–3 mm diameter) containing actively growing mycelium were transferred into 250 mL Erlenmeyer flasks containing 100 mL liquid PDA and incubated in the dark at 28 °C with shaking at 150 rpm for 7–14 days. Mycelia were harvested, washed three times with sterile ultrapure water, and resuspended at 2:1000 (*w*/*v*; 2 g fresh mycelium in 1000 mL water) to generate a *P. indica* inoculum as described previously [31]. The final inoculum concentration was adjusted to 2 × 10^7^ mycelial fragments (fresh weight equivalent). For inoculation, 30 pots each of colonized and control plants were selected, and 10 mL of the mycelial suspension was injected into the root zone of each pot at 3 and 5 days after the start of inoculation. Control plants received the same volume of sterile ultrapure water.

At 10 and 20 days after inoculation (dais), both colonized and control plants seedlings were harvested, rinsed, and used to measure plant height (cm), primary root length (cm), and root fresh and dry weight (g). Roots were dried at 55 °C to constant weight to determine dry biomass. For downstream analyses, entire root systems from six plants (three pots) were pooled as one biological replicate to minimize the impact of individual plant variation and to obtain a representative average molecular profile for the treatment group, which is essential for both physiological and molecular assays. All samples were immediately frozen in liquid nitrogen for 30 min and stored at −80 °C until physiological assays, transcriptome sequencing, and metabolomic profiling. Three biological replicates were used for all physiological and transcriptomic analysis, and four biological replicates were used for metabolomic analysis.

### 2.2. Fungal Colonization Assessment

Successful colonization was verified at 10 dais by microscopic examination of fungal structures in root cortical tissues. Briefly, 8–10 root samples from colonized and control plants were randomly collected, cleared in 10% (*w*/*v*) KOH in a water bath for 1 h, rinsed three times with ddH_2_O, stained with 0.04% Trypan Blue for 2 h, and washed three additional times with ddH_2_O. Fungal colonization was observed using an Olympus BX53 fluorescence microscope (Olympus, Shanghai, China).

### 2.3. Plant Physiological Trait Analysis

The same fresh root samples were used to quantify malondialdehyde (MDA) content and the activities of catalase (CAT), peroxidase (POD), and superoxide dismutase (SOD) using commercial assay kits (Beijing Solarbio Science & Technology Co., Ltd., Beijing, China) following the manufacturer’s protocols. Indole-3-acetic acid (IAA), jasmonic acid (JA), and 1-aminocyclopropane-1-carboxylic acid (ACC) contents were quantified using enzyme-linked immunosorbent assays (ELISA) kits (Beijing Solarbio Science & Technology Co., Ltd.) as per the manufacturer’s instructions, with the assay detecting total hormone levels.

All growth and physiological data were analyzed using SPSS 17.0 (SPSS Inc., Chicago, IL, USA). Data are presented as the mean ± standard deviation (SD) of three independent biological replicates. Statistical differences were assessed using Student’s *t*-test after confirming normality and homogeneity of variance. Significance levels are denoted as follows: *p* < 0.05 (*), *p* < 0.01 (**), *p* < 0.001 (***), and ns indicates *p* > 0.05.

### 2.4. Table Transcriptome Assembly and Functional Annotation

Total RNA was extracted from root tissues (1 µg RNA per sample input for library preparation) with three biological replicates per treatment using TRIzol reagent (Invitrogen, Carlsbad, CA, USA) according to the manufacturer’s instructions. RNA integrity and concentration were evaluated using an Agilent 2100 Bioanalyzer (Agilent Technologies, Palo Alto, CA, USA). Sequencing libraries were prepared using the NEBNext^®^ Ultra™ RNA Library Prep Kit for Illumina^®^ (NEB, Ipswich, MA, USA) following the manufacturer’s recommendations, and index codes were added to label each sample. Cluster generation was performed on a cBot Cluster Generation System using the TruSeq PE Cluster Kit v3-cBot-HS (Illumina, San Diego, CA, USA). Libraries were sequenced on an Illumina HiSeq platform to generate 125/150 bp paired-end reads. Quality control was conducted using in-house R scripts to remove adapter sequences and low-quality bases. Clean reads were obtained by discarding reads containing ambiguous bases (“N”) and those where >50% of bases had a Phred quality score ≤ 10. Subsequently, quality metrics including Q30, GC content, and sequence duplication levels were calculated for the cleaned datasets. Clean reads were assembled de novo using Trinity (v2.4.0) [32], a widely adopted and efficient tool specifically designed for reconstructing full-length transcripts from RNA-Seq data in the absence of a reference genome. For each Trinity “gene”, the longest transcript was selected as the unigene for downstream analyses. Unigenes were annotated against the following databases: NCBI non-redundant protein (Nr), Gene Ontology (GO), Kyoto Encyclopedia of Genes and Genomes (KEGG) [33], Swiss-Prot, eggNOG, Pfam, and clusters of orthologous group for eukaryotic complete (KOG).

### 2.5. Gene Expression Quantification and DEG Identification

Clean reads from each library were aligned back to the assembled unigene set, and gene-level abundances were estimated using RSEM [34]. RSEM-generated expected read counts were used as input for differential expression analysis. Expression values were additionally reported as fragments per kilobase of transcript per million mapped reads (FPKMs) for visualization purposes [35]. Differential expression analysis was performed using the DESeq R package (v1.10.1). To analyze the global gene reprogramming trend, we performed K-means clustering on Z-score-standardized expression values of all detected unigenes. PCAs were performed using the BMK Cloud platform (www.biocloud.net, accessed on 24 October 2025). *p* values were adjusted using the Benjamini–Hochberg method to control the false discovery rate (FDR). Genes with a fold change (FC) ≥ 2 and FDR < 0.05 were defined as differentially expressed genes (DEGs). GO enrichment was conducted using the topGO R package (v1.10.1), and KEGG enrichment was performed with KOBAS [36]. DEG expression patterns were visualized using row Z-score-standardized heat maps. Transcription factors (TFs) among DEGs were annotated using BMKCloud (www.biocloud.net, accessed on 24 October 2025).

### 2.6. qRT-PCR Validation

qRT-PCR was performed using SYBR Green qPCR Mix (TIANGEN, Beijing, China) on an iQ5 Multicolor Real-Time PCR Detection System (Bio-Rad, Hercules, CA, USA). The cycling program was 95 °C for 15 min, followed by 40 cycles of 95 °C for 10 s and 60 °C for 30 s. Relative expression levels were calculated using the 2^−ΔΔCt^ method [37]. *Actin7* (unigene ID: TRINITY_DN2878_c0_g1) was used as the internal reference. Primer sequences are listed in Table S1. The correlation between RNA-seq (FPKMs) and qRT-PCR data was assessed by linear regression. Each data point represents the mean expression level from three biological replicates.

### 2.7. Untargeted Metabolomic Profiling

Sample preparation, UPLC-MS/MS analysis, metabolite identification, and quantification were performed by Biomarker Technologies Co., Ltd. (Beijing, China) following the procedures described by Chen et al. [38]. Metabolites were annotated using the KEGG compound database and mapped to KEGG pathways. The *R* language package (v1.10.1) ROPL was utilized for OPLS-DA modeling. Two hundred permutation tests were conducted to verify the reliability of the model. The variable importance in the projection (VIP) value of the model was calculated by multiple cross-validations. To analyze the changing trend of metabolites, we performed clustering on all detected metabolites after Z-score standardization. PCAs were performed using the BMK Cloud platform (www.biocloud.net, accessed on 24 October 2025). The differential metabolites of the orthogonal partial least-squares discriminant analysis (OPLS-DA) model were screened by combining the difference multiple, *p* value, and VIP value. Metabolites with VIP ≥ 1, *p* < 0.05, and |Log_2_FC| ≥ 1.5 were defined as differentially accumulated metabolites (DAMs). The DAM analysis followed a workflow analogous to that of DEGs.

### 2.8. Integrated Transcriptomic and Metabolomic Analyses

DEGs and DAMs from the same comparisons were jointly mapped to KEGG pathways. To identify potential regulatory relationships, Pearson correlation coefficients between genes and metabolites were calculated in R, and association networks were visualized using edges with PCC > 0.8. These correlations represent statistical associations and do not imply direct causation. In addition, O2PLS models were constructed using all DEGs and DAMs. Variables with high loadings and strong cross-dataset contributions in the O2PLS model were prioritized as key features potentially influencing or reflecting changes in the other omics layer [39].

## 3. Results

### 3.1. Growth and Physiological Responses of B. Decumbens to P. indica Colonization

**Figure 1:**
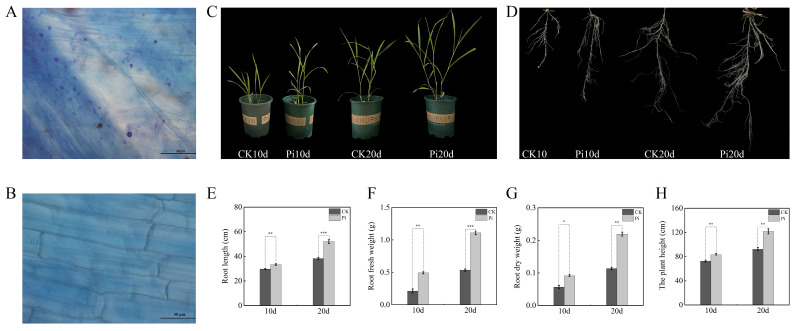
Phenotypic observation and morphological characteristics of *B. decumbens* seedlings inoculated with *P. indica*. (**A**) *P. indica*-colonized *B. decumbens* root cells under a microscope. (**B**) Control *B. decumbens* root cells under a microscope. (**C**) Typical control (CK) and *P. indica*-colonized *B. decumbens* seedlings. (**D**) Typical roots of control (CK) and *P. indica*-colonized *B. decumbens* seedlings. (**E**–**H**) Root length, root fresh weight, root dry weight, and plant height of control (CK) and *P. indica*-colonized *B. decumbens* seedlings. Three independent biological replicates were used to calculate the means. Error bars indicate the standard deviation (SD). Values indicate means ± SD. Student’s *t*-test was applied to verify the significant differences among treatments. *, *p* < 0.05; **, *p* < 0.01; ***, *p* < 0.001.

Successful colonization of *P. indica* in *B. decumbens* roots at 10 days after inoculation (dais) was confirmed by Trypan Blue staining, which revealed characteristic fungal structures in cortical tissues (Figure 1A,B). Inoculated cuttings displayed enhanced growth relative to mock controls, with visibly increased plant height and root length (Figure 1C,D). Quantitatively, *P. indica* colonization significantly increased primary root length by 22.34% (*p* < 0.01) at 10 dais and 56.16% (*p* < 0.001) at 20 dais compared with the corresponding controls (Figure 1E). Root biomass was also elevated: root fresh weight significantly increased by 85.22% (*p* < 0.01) and 107.22% (*p* < 0.001) at 10 and 20 dais, respectively, while root dry weight significantly increased by 62.35% (*p* < 0.05) and 93.53% (*p* < 0.01) at the two time points (Figure 1F,G). Plant height was similarly higher in colonized seedlings (Figure 1H).

**Figure 2:**
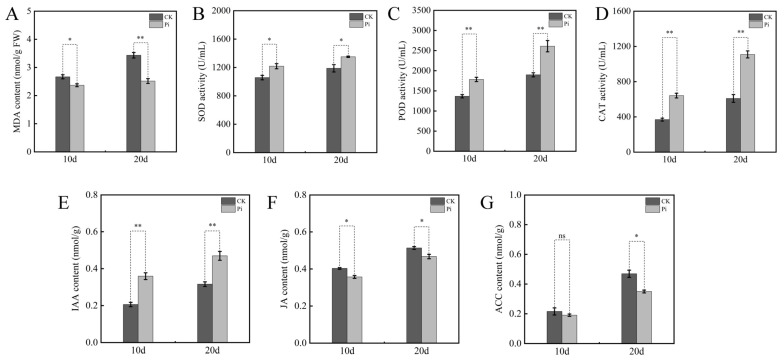
Physiological responses of *B. decumbens* roots under *P. indica* colonization. (**A**) Malondialdehyde content; (**B**) superoxide dismutase activity; (**C**) peroxidase activity; (**D**) catalase activity; (**E**) indole-3-acetic acid; (**F**) jasmonic acid; (**G**) 1-aminocyclopropane-1-carboxylic acid. Three independent biological replicates were used to calculate the means. Error bars indicate the standard deviation (SD). Values indicate means ± SD. Student’s *t*-test was applied to verify the significant differences among treatments. *, *p* < 0.05; **, *p* < 0.01; *p* > 0.05, ns.

We next assessed oxidative status and antioxidant capacity in roots during colonization. Compared with the controls, inoculated roots at both stages showed lower malondialdehyde (MDA) levels together with higher activities of superoxide dismutase (SOD), peroxidase (POD), and catalase (CAT) (Figure 2A–D), consistent with reduced lipid peroxidation and strengthened antioxidant capacity. Phytohormone-related profiling further indicated marked hormonal shifts. Indole-3-acetic acid (IAA) significantly increased in colonized roots, reaching 74.42% (*p* < 0.01) and 48.51% (*p* < 0.01) above mock controls at 10 and 20 dais, respectively (Figure 2E). In contrast, jasmonic acid (JA) was markedly reduced (*p* < 0.05) to 11.44% (10 dais) and 9.52% (20 dais) of the corresponding control levels (Figure 2F). The ethylene precursor 1-aminocyclopropane-1-carboxylic acid (ACC) changed only slightly at 10 dais (*p* > 0.05) but declined significantly at 20 dais to 25.44% (*p* < 0.05) of the control (Figure 2G). Because growth promotion and associated physiological changes were already evident at 10 dais and were more pronounced at 20 dais, these two stages were selected for transcriptomic and metabolomic analyses.

### 3.2. Overview of Transcriptome Sequencing and Assembly

Twelve cDNA libraries were constructed, including three biological replicates each for Pi10d and Pi20d and their corresponding mock controls (CK10d and CK20d; three replicates per time point). Raw reads were deposited in NCBI under accession PRJNA1372385. After removing low-quality reads, adaptor sequences, and poly-N reads, 20.55–26.26 million clean reads were retained per library, totaling 81.94 Gb of clean bases. Q30 values ranged from 96.37% to 97.47%, and GC contents ranged from 52.22% to 54.15% (Table S2). Clean reads from all libraries were assembled de novo using Trinity, generating 99,996 unigenes with a mean length of 711.97 bp (Table S3). Clean reads from each library were then aligned back to the assembled unigene set using Bowtie2 to support downstream expression quantification. Expression summaries for all unigenes are provided in Table S4.

**Figure 3:**
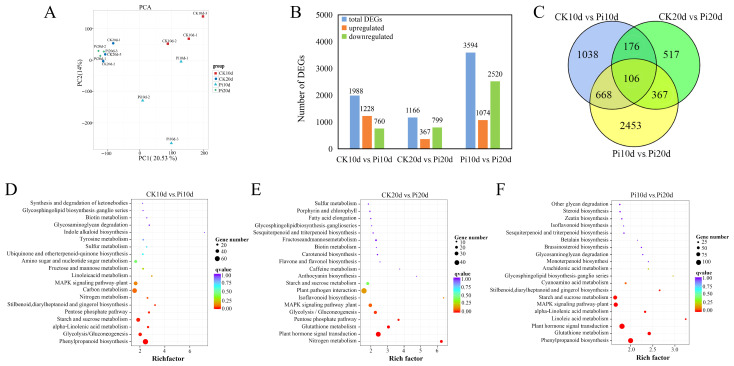
Transcriptomic profiling of *B. decumbens* roots under *P. indica* colonization. (**A**) PCA of DEGs in three comparisons, Pi10d vs. CK10d, Pi20d vs. CK20d, and Pi20d vs. Pi10d. (**B**) The number of differentially expressed genes (DEGs) between each group in the pairwise comparison. (**C**) Venn diagrams showing the overlap among DEGs in each of the different treatments. (**D**) KEGG enrichment analysis of the DEGs in the Pi10d vs. CK10d group. (**E**) KEGG enrichment analysis of the DEGs in the Pi20d vs. CK20d group. (**F**) KEGG enrichment analysis of the DEGs in the Pi20d vs. Pi10d group.

Principal component analysis (PCA) showed clear separation among sample groups (Figure 3A), indicating that *P. indica* colonization was associated with substantial transcriptional reprogramming. Treatment and control samples were well separated at 10 dais, whereas their distributions were closer at 20 dais, consistent with stage-dependent modulation of host responses during symbiosis establishment. To characterize expression dynamics across sample groups, all unigenes were subjected to K-means clustering, yielding nine clusters with distinct temporal patterns (Figure S1). Cluster 6 contained 4662 unigenes that were induced at both 10 and 20 dais, suggesting a sustained transcriptional program associated with *P. indica* colonization. To validate RNA-seq results, ten unigenes were examined by qRT-PCR (Table S1), and expression trends were compared with RNA-seq estimates (Figure S2A). Linear regression showed strong concordance between the two methods (R^2^ = 0.9107; Figure S2B), supporting the reliability of the transcriptome dataset. For functional annotation, unigenes were searched against multiple databases, including NR, NT, Swiss-Prot, GO, KEGG, Pfam, eggNOG, and KOG. In total, 62,755 unigenes were annotated in at least one database (Table S5). NR contributed the largest number of annotations, followed by GO and eggNOG/Swiss-Prot (Table S5).

### 3.3. Differential Gene Expression in Response to P. indica Colonization

Differential expression analysis was conducted for three pairwise comparisons: Pi10d vs. CK10d, Pi20d vs. CK20d, and Pi20d vs. Pi10d. Using the thresholds of FC ≥ 2 and FDR < 0.05, we identified 1988 DEGs (1228 upregulated and 760 downregulated) in Pi10d vs. CK10d, 1166 DEGs (367 upregulated and 799 downregulated) in Pi20d vs. CK20d, and 3594 DEGs (1074 upregulated and 2520 downregulated) in Pi20d vs. Pi10d (Figure 3B; Tables S6–S8). Venn analysis indicated that 106 DEGs were shared across all three comparisons, whereas 1038 and 517 DEGs were specific to Pi10d vs. CK10d and Pi20d vs. CK20d, respectively (Figure 3C). For example, TRINITY_DN17338_c0_g1 (annotated as a hypothetical protein PAHAL) functions in the biosynthesis of cell walls, membranes, and envelopes. Similarly, genes such as TRINITY_DN25452_c0_g4 (annotated as dehydrin DHN3) and TRINITY_DN9730_c0_g1 (annotated as zinc finger protein ZAT1) are associated with secondary metabolism as well as amino acid and lipid transport and metabolism (Tables S6–S8). All of these co-upregulated genes represent crucial candidates for subsequent functional studies and molecular breeding.

GO and KEGG enrichment analyses were performed to infer DEG-associated functions. Across Pi10d vs. CK10d, Pi20d vs. CK20d, and Pi20d vs. Pi10d, 1515, 875, and 2756 DEGs, respectively, were assigned to at least one GO term in biological process (BP), molecular function (MF), or cellular component (CC) categories (Figure S3). In Pi10d vs. CK10d and Pi20d vs. Pi10d, enriched terms included hydrogen peroxide catabolic processes, responses to oxidative stress, and carbohydrate metabolism. In Pi20d vs. CK20d, DEGs were preferentially associated with fructose 6-phosphate metabolism and glutathione-related processes, indicating stage-dependent metabolic adjustments (Figure S3). KEGG enrichment further highlighted pathways underlying early and late responses (Figure 3D–F; Table S9). In Pi10d vs. CK10d, the most significantly enriched pathways included phenylpropanoid biosynthesis (ko00940), glycolysis/gluconeogenesis (ko00010), α-linolenic acid metabolism (ko00592), and starch and sucrose metabolism (ko00500) (Figure 3D). In Pi20d vs. CK20d, nitrogen metabolism (ko00910), plant hormone signal transduction (ko04075), glutathione metabolism (ko00480), and the pentose phosphate pathway (ko00030) were enriched (Figure 3E). In Pi20d vs. Pi10d, phenylpropanoid biosynthesis (ko00940), glutathione metabolism (ko00480), plant hormone signal transduction (ko04075), and linoleic acid metabolism (ko00591) were significantly enriched (Figure 3F). Together, these results indicate that *P. indica* colonization is associated with dynamic transcriptional reprogramming in *B. decumbens* roots, with pathway priorities differing between early and later stages.

### 3.4. Analysis of Transcription Factors

Transcription factors (TFs) are key regulators of gene expression and are frequently reprogrammed during beneficial plant–microbe interactions. Based on RNA-seq, we identified 99 TF-related DEGs (41 upregulated and 58 downregulated) in Pi10d vs. CK10d, 104 TF-related DEGs (35 upregulated and 69 downregulated) in Pi20d vs. CK20d, and 222 TF-related DEGs (68 upregulated and 154 downregulated) in Pi20d vs. Pi10d (Figure S4; Table S10). These TFs spanned multiple families, including AP2/ERF, NAC, Tify, C2H2, bHLH, MYB, and WRKY, indicating broad transcriptional remodeling during colonization. Notably, a subset of TFs showed consistent induction in both colonization comparisons (Pi10d vs. CK10d and Pi20d vs. CK20d) and higher expression at 20 dais relative to 10 dais in colonized roots (Pi20d vs. Pi10d), suggesting progressive activation during symbiosis establishment. These included members of bZIP, AP2/ERF, NAC, MYB, bHLH, GARP-G2, and HB-other families (Table S10). Transcription factors (TFs) orchestrate a wide array of biological processes in plants. For example, NAC TFs, representing a large plant-specific family, are crucial for hormone signal transduction, growth regulation, and stress resistance [40]. bHLH TFs are potential stress-response regulators involved in biosynthesis through the phenylpropanoid pathway; their numerous members suggest significant roles in plant physiology [40,41]. AP2/ERF TFs are associated with secondary metabolite biosynthesis and stress response [42], and MYB TFs, involved in anthocyanin synthesis, appear to participate in both biotic and abiotic processes [40,43]. These TFs are thus hypothesized to play pivotal roles in mediating the growth-promoting effects and symbiotic establishment within the *B. decumbens*–*P. indica* interaction.

### 3.5. Metabolic Profiling of B. decumbens Roots in Response to P. indica Colonization

**Figure 4:**
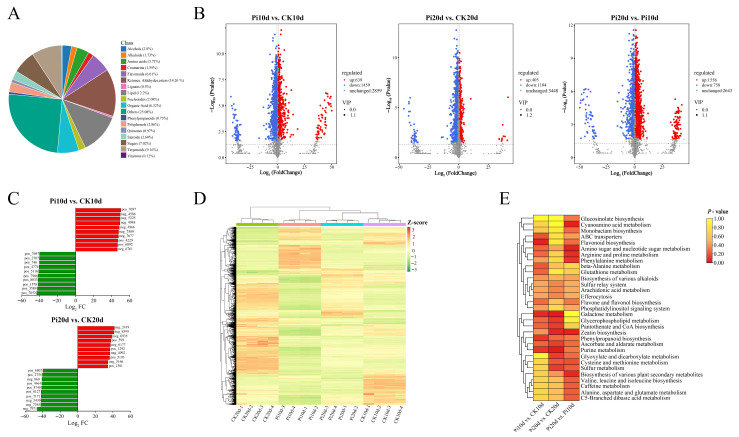
Metabolic profiling of *B. decumbens* roots under *P. indica* colonization. (**A**) Classifications of total 4957 metabolites identified with UPLC-MS/MS. (**B**) Volcano plot of differentially accumulated metabolites (DAMs) in three comparisons, Pi10d vs. CK10d, Pi20d vs. CK20d, and Pi20d vs. Pi10d. (**C**) Top 10 up- and downregulated DAMs in Pi10d vs. CK10d and Pi20d vs. CK20d, respectively. (**D**) Clustering analysis heat map of the expression of DAMs detected in each sample. The color scale indicates the relative levels of metabolites: red indicates upregulated metabolites, while green indicates downregulated metabolites. (**E**) The KEGG pathway analysis of the DAMs based on *p*-values.

To obtain a global view of metabolic reprogramming in *B. decumbens* roots following *P. indica* colonization, we performed untargeted metabolomic profiling using a UPLC-MS/MS platform. After annotation, 4957 compounds were detected across all samples, spanning diverse chemical classes, including aldehydes/ketones/esters (14.26%), lipids (12.20%), terpenoids (9.16%), sugars (7.02%), and flavonoids (6.01%) (Figure 4A; Table S11). PCA showed clear separations driven by colonization status and sampling time and indicated good replicate consistency (Figure S5). Z-score-normalized abundances were further examined by hierarchical clustering (Figure 4C; Figure S6). Orthogonal partial least-squares discriminant analysis (OPLS-DA) was performed for each comparison, and colonized versus control samples were clearly separated (Figure S7A–C). Permutation-based validation suggested that the models were not overfitted for both ion modes (Figure S7D–F).

DAMs were defined by |Log_2_FC| ≥ 1.5, VIP > 1.0, and *p* < 0.05, yielding 2098 DAMs (639 upregulated and 1459 downregulated) in Pi10d vs. CK10d, 1509 DAMs (405 upregulated and 1104 downregulated) in Pi20d vs. CK20d, and 2314 DAMs (1556 upregulated and 758 downregulated) in Pi20d vs. Pi10d (Figure 4B; Tables S12–S14). K-means clustering categorized DAMs into nine clusters with distinct accumulation patterns (Figure S8). The ten most strongly altered metabolites in each comparison are shown in Figure 4D. In Pi10d vs. CK10d, the top upregulated DAMs were dominated by phenolic compounds, whereas the top downregulated metabolites were largely lipids. In Pi20d vs. CK20d, the top upregulated DAMs were mainly carbohydrates and nucleotide derivatives, while downregulated DAMs were enriched in nucleotide derivatives and phenylpropanoids. In Pi20d vs. Pi10d, the top upregulated DAMs again largely comprised phenolic compounds, whereas the most strongly downregulated DAMs were primarily lipids. Overall, metabolite remodeling during colonization was characterized by prominent shifts involving lipids, carbohydrates, and phenolic-related compounds.

KEGG enrichment of DAMs (*p* < 0.05) highlighted stage-dependent metabolic responses (Figure 4E; Table S15). DAMs in Pi10d vs. CK10d were enriched in flavonoid biosynthesis (ko00941). In Pi20d vs. CK20d, galactose metabolism (ko00052) and purine metabolism (ko00230) were enriched. In Pi20d vs. Pi10d, cyanoamino acid metabolism (ko00460) and zeatin biosynthesis (ko00908) were enriched (Figure 4E; Table S15). Together, these results indicate that *P. indica* colonization is accompanied by broad and stage-dependent metabolic reprogramming in *B. decumbens* roots.

### 3.6. Integrated Transcriptomic and Metabolomic Analysis of the Response of B. decumbens Roots to P. indica Colonization

**Figure 5:**
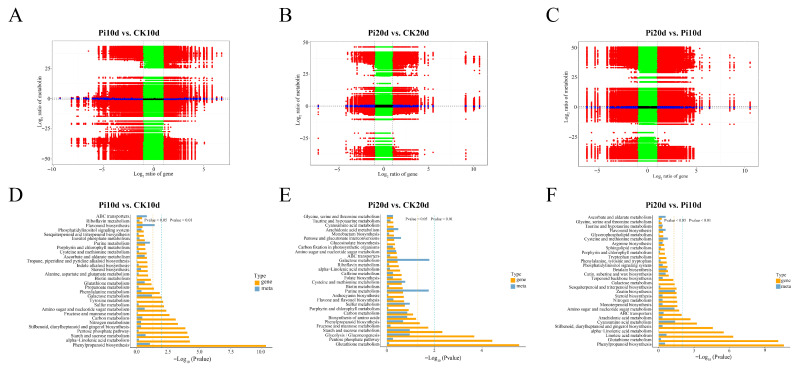
Integrated analysis of DEGs and DAMs. (**A**–**C**) Quadrant diagrams representing the association of the DAMs and DEGs in three comparisons. Horizontal coordinates are the multiplicity of differences in genes; vertical coordinates are the multiplicity of differences in metabolites. Black: differentially grouped genes and metabolites that are not differentially expressed. Red and green: unchanged metabolites, up- and downregulated genes, or unchanged genes with up- and downregulated metabolites. Left diagonal: genes and metabolites with consistent regulatory trends; changes in metabolites may be positively regulated by genes. Right diagonal: genes and metabolites with non-consistent regulatory trends; changes in metabolites may be negatively regulated by genes. (**D**–**F**) KEGG pathway of DEGs and DAMs in three comparisons. Vertical coordinates are metabolic pathway names, horizontal coordinates are *p* values from enrichment analyses of two histologies. Colors represent different histologies: yellow dashed line is *p* = 0.05 position and above the dashed line is the result of *p* < 0.05; blue dashed line is *p* = 0.01 position and above the dashed line is the result of *p* < 0.01.

To explore potential linkages between transcriptional changes and metabolite accumulation, we performed an integrated analysis of the transcriptomic and metabolomic datasets by assessing statistical associations between DEGs and DAMs. DEGs (FDR < 0.05) and DAMs (VIP > 1, *p* < 0.05) were subjected to pairwise correlation analysis across all biological replicates. Gene–metabolite pairs with |PCC| > 0.8 were considered to represent strong statistical associations for network inference. It should be emphasized that these correlations indicate coordinated changes rather than direct causal relationships. Nine-quadrant plots showed that discordant DEG-DAM patterns were more frequent than concordant patterns across the three comparisons (Figure 5A–C). This prevalence of discordant trends is not unexpected in biological systems, as it can arise from pathway branching, post-translational regulation, differential metabolite turnover rates, or the involvement of the measured metabolites in multiple interconnected pathways. Thus, these patterns reflect the complex, often indirect relationships between transcript abundance and metabolite levels.

KEGG co-enrichment analysis revealed 58, 49, and 64 pathways jointly enriched by DEGs and DAMs in Pi10d vs. CK10d, Pi20d vs. CK20d, and Pi20d vs. Pi10d, respectively (Figure 5D–F; Table S16). In Pi10d vs. CK10d, co-enriched pathways included phenylpropanoid biosynthesis (ko00940), α-linolenic acid metabolism (ko00592), starch and sucrose metabolism (ko00500), and the pentose phosphate pathway (ko00030). In Pi20d vs. CK20d, co-enriched pathways included biosynthesis of amino acids (ko01230), monobactam biosynthesis (ko00261), glucosinolate biosynthesis (ko00966), cyanoamino acid metabolism (ko00460), and phenylpropanoid biosynthesis (ko00940). In Pi20d vs. Pi10d, co-enriched pathways included glycine, serine, and threonine metabolism (ko00260), inositol phosphate metabolism (ko00562), carbon metabolism (ko01200), and flavone and flavonol biosynthesis (ko00944) (Figure 5D–F; Table S16). These results suggest coordinated, yet stage-dependent, shifts in gene expression and metabolite accumulation during colonization.

### 3.7. Coordinated DEG and DAM Changes in Phenylpropanoid Biosynthesis

**Figure 6:**
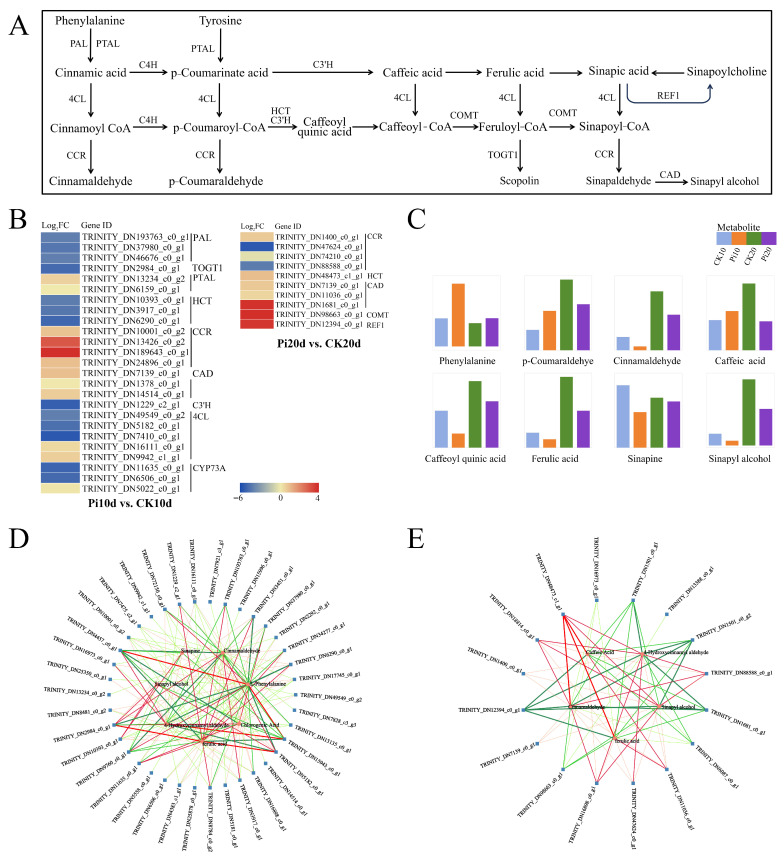
Analysis of the DEGs and DMs associated with phenylpropanoid biosynthesis in *B. decumbens* roots under *P. indica* colonization. (**A**) Phenylpropanoid biosynthesis pathway. CAD, cinnamyl alcohol dehydrogenase; CYP73A, trans-cinnamate 4-monooxygenase; 4CL, 4-coumarate-CoA ligase; CCR, cinnamoyl-CoA reductase; CH’3, p-coumarate 3-hydroxylase; REF1, coniferyl-aldehyde dehydrogenase; PAL, phenylalanine ammonia-lyase; PTAL, phenylalanine/tyrosine ammonia-lyase; HCT, shikimate O-hydroxycinnamoyltransferase. (**B**) The heat map diagram of expression levels of DEGs involved in phenylpropanoid biosynthesis was generated based on the value of Log_2_ Fold Change. The red color represents a high level of expression and green represents low. (**C**) Relative expression of key metabolites related to phenylpropanoid biosynthesis. (**D**) Correlation network of the DEGs and DAMs involved in phenylpropanoid biosynthesis in Pi10d vs. CK10d. (**E**) Correlation network of the DEGs and DAMs involved in phenylpropanoid biosynthesis in Pi20d vs. CK20d. Red circles indicate metabolites and blue squares indicate genes. Red lines and green lines indicate positive and negative regulatory relationships between genes and metabolites, respectively.

Given the consistent co-enrichment signals, we further examined phenylpropanoid biosynthesis as a candidate response pathway during *P. indica* colonization. KEGG-based mapping identified 25 DEGs in Pi10d vs. CK10d and 10 DEGs in Pi20d vs. CK20d, including 12 and 8 upregulated genes, respectively (Figure 6A,B). Several lignin/phenylpropanoid-associated enzyme families showed increased expression at the early stage, including cinnamyl alcohol dehydrogenase (CAD), cinnamoyl-CoA reductase (CCR), phenylalanine/tyrosine ammonia-lyase (PTAL), and p-coumarate 3-hydroxylase (C3′H) (Figure 6B). At 20 dais, caffeoyl-CoA 3-O-methyltransferase (COMT), cinnamyl alcohol dehydrogenase (CAD), shikimate O-hydroxycinnamoyltransferase (HCT), and coniferyl-aldehyde dehydrogenase (REF1) family genes were induced (Figure 6B), consistent with modulation of cell wall-associated phenylpropanoid branches. Eight phenylpropanoid-related metabolites were detected, including L-phenylalanine, p-coumaraldehyde, cinnamaldehyde, caffeoyl quinic acid, ferulic acid, sinapoylcholine, sinapyl alcohol, and caffeic acid (Figure 6C). L-phenylalanine and p-coumaraldehyde were upregulated, whereas several downstream metabolites showed reduced accumulation (Figure 6C). PCC-based networks revealed strong positive correlations (PCC > 0.8) between 38 DEGs and seven DAMs in Pi10d vs. CK10d and between 16 DEGs and five DAMs in Pi20d vs. CK20d (Figure 6D,E). These high correlations are indicative of a potential co-regulatory relationship between gene expression and metabolite accumulation in the phenylpropanoid pathway across different colonization stages.

### 3.8. Coordinated DEG and DAM Changes in α-Linolenic Acid Metabolism

**Figure 7:**
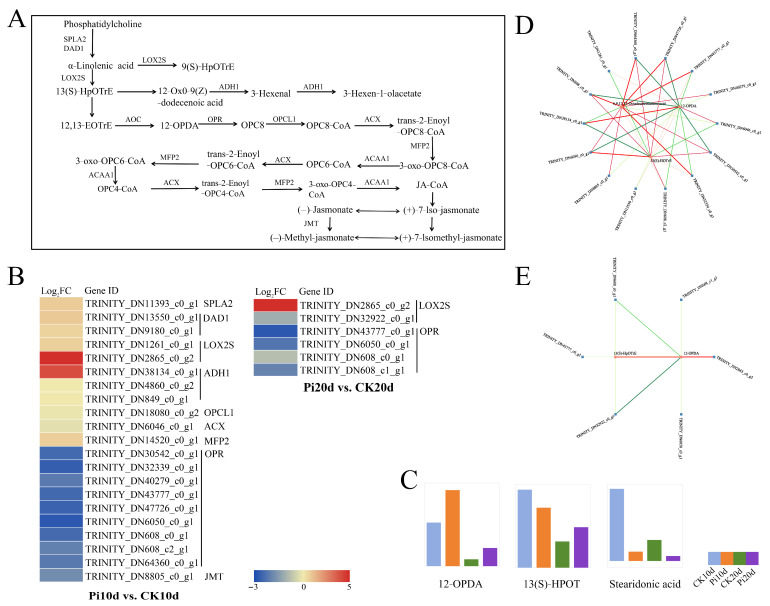
Analysis of the DEGs and DMs associated with α-Linolenic acid metabolism in *B. decumbens* roots under *P. indica* colonization. (**A**) α-Linolenic acid metabolism pathway. SPLA2, secretory phospholipase A2; DAD1, phospholipase A1; LOX2S, lipoxygenase; ADH1, alcohol dehydrogenase; OPCL1, OPC-8:0 CoA ligase 1; ACX, acyl-CoA oxidase; MFP2 enoyl-CoA hydratase; OPR, 12-oxophytodienoic acid reductase; JMT, jasmonate O-methyltransferase; ACO, acetyl-CoA acyltransferase 1; ACAA1, acetyl-CoA acyltransferase 1. (**B**) The heat map diagram of expression levels of DEGs involved in alpha-Linolenic acid metabolism was generated based on the value of Log_2_ Fold Change. The red color represents a high level of expression and green represents low. (**C**) Relative expression of key metabolites related to α-linolenic acid metabolism. (**D**) Correlation network of the DEGs and DAMs involved in α-Linolenic acid metabolism in Pi10d vs. CK10d. (**E**) Correlation network of the DEGs and DAMs involved in α-Linolenic acid metabolism in Pi20d vs. CK20d. Red circles indicate metabolites and blue squares indicate genes. Red lines and green lines indicate positive and negative regulatory relationships between genes and metabolites, respectively.

α-Linolenic acid metabolism was also highlighted by joint enrichment analysis (Figure 7A). Within this pathway, 15 DEGs were identified in Pi10d vs. CK10d and 6 DEGs in Pi20d vs. CK20d (Figure 7B). These genes encoded enzymes involved in lipid remodeling and oxylipin-related reactions, including phospholipase A1 (DAD1-like), OPC-8:0 CoA ligase (OPCL1-like), lipoxygenase (LOX-like), alcohol dehydrogenase (ADH-like), acyl-CoA oxidase (ACX-like), secretory phospholipase A2 (SPLA2-like), and enoyl-CoA hydratase (MFP2-like) (Figure 7B).

Three metabolites within this pathway were detected, including 12-OPDA, 13(S)-HOTrE, and stearidonic acid (Figure 7C). Notably, 12-OPDA accumulated in colonized roots relative to controls (Figure 7C). Network analysis further revealed strong positive correlations (PCC > 0.8) between 14 DEGs and three DAMs in Pi10d vs. CK10d and between 6 DEGs and two DAMs in Pi20d vs. CK20d (Figure 7D,E). This implies that the dynamic changes in gene expression and metabolite abundance in the α-linolenic acid metabolism pathway during colonization could be linked through an underlying regulatory mechanism.

## 4. Discussion

*P. indica* is a beneficial root endophyte with a broad host range, and growth-promoting effects have been reported across diverse plant species [10,44,45,46,47,48]. It has been reported to colonize more than 200 plant species [5,47], and its axenic cultivability has made it a widely used model for studying beneficial plant–fungus interactions. Although *P. indica* has been examined in several grass and forage species, such as rice (*Oryza sativa*) [48], barley (*Hordeum vulgare*) [18,49], *Salvia leucantha* [50], and alfalfa (*Medicago sativa*) [51], its interaction with *B. decumbens* has not, to our knowledge, been reported. Here, Trypan Blue staining revealed clear fungal structures in *B. decumbens* roots at 10 dais, indicating that *P. indica* can colonize this host. Colonization was accompanied by increased plant height and enhanced root development, supporting the potential agronomic relevance of this interaction. The growth stimulation observed in colonized seedlings was accompanied by consistent changes in oxidative status. Antioxidant enzyme activities (SOD, POD, and CAT) were elevated following inoculation, whereas MDA content (a marker of lipid peroxidation) declined at both 10 and 20 dais. These patterns are consistent with improved redox homeostasis during compatible colonization. In particular, POD activity has been proposed as an indicator of rooting performance, and increased POD activity in *P. indica*-colonized Tartary buckwheat roots was associated with enhanced rooting ability [52]. In line with these findings, our results suggest that strengthened antioxidant capacity and reduced oxidative damage may contribute to root growth promotion in *B. decumbens*. In addition, MDA decreased by 11.25% and 26.70% in *B. decumbens* roots at 10 and 20 dais, respectively, relative to mock controls. This reduction aligns with previous studies reporting that *P. indica* colonization is frequently associated with improved redox homeostasis and reduced membrane lipid peroxidation in host roots under diverse contexts [45,52,53]. Consequently, these findings point toward practical applications for enhancing forage systems.

Phytohormone remodeling is a hallmark of root colonization by beneficial microbes. Jasmonates, including JA and its derivatives, mediate defense against pathogens and herbivores and also shape interactions with beneficial root-associated microorganisms [54]. JA responses to *P. indica* can be host- and context-dependent [16]. For example, Vahabi et al. [55] reported that direct exposure of *Arabidopsis* roots to *P. indica* hyphae increased JA and JA-Ile levels without abolishing growth benefits. In contrast, we observed pronounced suppression of JA in colonized *B. decumbens* roots. One plausible interpretation is that JA downregulation in *B. decumbens* reflects attenuation of defense-associated signaling during compatible colonization, which may favor establishment and maintenance of the interaction. JA signaling is closely coordinated with ethylene, whose biosynthesis depends on ACC [56,57]. In our study, ACC changed modestly at 10 dais but declined substantially at 20 dais, consistent with progressive modulation of JA-ethylene-associated outputs during symbiosis development. Similar JA downregulation has been reported in other systems, including *Dimocarpus longan* and *Cerasus humilis*, during *P. indica* colonization [15,52]. Auxin is a central regulator of root growth and is frequently implicated in symbiosis establishment [58]. Consistent with this, we detected increased IAA levels in colonized roots, together with reduced JA and ACC. Because auxin and jasmonate pathways can interact antagonistically or context-dependently, the observed hormonal profile suggests that *P. indica* colonization is associated with hormonal rebalancing-enhancing auxin-linked developmental signaling while dampening JA/ethylene-associated defense outputs—similar to patterns reported in other *P. indica*–host systems [57,59].

Beyond physiological readouts, multi-omics approaches enable pathway-level interpretation of plant–microbe interactions [60,61,62]. Here, integrated transcriptomic and metabolomic profiling revealed substantial remodeling of core metabolisms across colonization stages. DEGs were enriched in pathways related to central carbon metabolism (e.g., glycolysis/gluconeogenesis and the pentose phosphate pathway) and nitrogen metabolism, whereas DAMs were enriched in secondary metabolism and carbohydrate-related pathways. Similar signatures, including shifts in amino acid and lipid metabolism, have been reported during *P. indica* colonization in other hosts [12,19], supporting the generality of metabolic reprogramming during compatible symbiosis.

Secondary metabolism, particularly the phenylpropanoid pathway, has repeatedly been implicated in plant responses to beneficial microbes and endophytes [63,64,65]. Our integrated analysis highlighted phenylpropanoid biosynthesis as a consistently responsive pathway during *P. indica* colonization. This pathway generates phenolic acids, lignin-related monomers, and flavonoids, with phenylalanine and tyrosine serving as key precursors [66,67]. At the early stage (Pi10d), we observed induction of multiple phenylpropanoid/lignin-associated genes (e.g., PTAL, C3′H, CCR, and CAD), together with increased abundance of L-phenylalanine. This pattern is consistent with enhanced engagement of phenylpropanoid branches that may support root structural remodeling and/or defense-associated adjustments during colonization. An interesting feature was the divergence between PAL and PTAL expression. Although PAL is often regarded as a key entry enzyme into the general phenylpropanoid pathway, PAL was downregulated in our dataset, whereas PTAL and several lignin-associated genes were induced. This pattern may reflect grass-specific routing, as bifunctional PTALs can channel both phenylalanine and tyrosine into p-coumarate and downstream lignin-related metabolism [68]. PTAL activity depends on the conserved MIO (4-methylidene-imidazole-5-one) group in the active site [69], which may enable flux redistribution under specific contexts. Notably, despite transcriptional induction of phenylpropanoid genes, several downstream metabolites decreased in abundance. Such gene–metabolite discordance can arise from branch competition and isoenzyme specificity, transport, and turnover, and therefore, transcript abundance may not translate directly into steady-state metabolite levels [66]. Together, these results suggest that *P. indica* colonization reshapes phenylpropanoid-related regulation in *B. decumbens* roots through coordinated but non-linear changes at transcript and metabolite levels.

Lipid metabolism, particularly α-linolenic acid-derived pathways, also emerged as a major responsive module. α-linolenic acid is a precursor for oxylipins, including 12-OPDA and JA, which are involved in growth-defense coordination and stress responses [70,71,72,73]. In our study, multiple α-linolenic acid pathway genes (e.g., LOX-like, ACX-like, and OPCL1-like) and 12-OPDA showed induction/accumulation during early colonization, suggesting activation of lipid-derived metabolism during interaction establishment. Similar lipid rewiring has been reported in tomato, where *P. indica* colonization upregulated divergent fatty acid desaturases potentially linked to specialized metabolites [11]. This parallel raises the possibility that *P. indica* may reconfigure lipid-associated networks in *B. decumbens* beyond hormone biosynthesis. A notable outcome was that JA levels declined despite increased 12-OPDA accumulation and activation of α-linolenic acid-related genes. This suggests that JA steady-state levels may be shaped not only by biosynthesis but also by conversion, conjugation, and catabolism. One hypothesis is enhanced cytochrome P450-mediated oxidation of JA-Ile, a negative feedback route that attenuates jasmonate signaling. Aubert et al. [74] showed that overexpression of CYP94B3 or CYP94C1 increased oxidized JA-Ile derivatives (e.g., 12-OH-JA-Ile and 12-COOH-JA-Ile), leading to suppressed defense outputs. Future work quantifying JA-Ile and its oxidized derivatives and examining CYP94-family expression/activity will help test whether accelerated JA-Ile turnover contributes to JA reduction in colonized *B. decumbens* roots.

Taken together, our findings not only elucidate the molecular mechanisms underpinning this symbiotic interaction but also highlight its practical potential. Specifically, *P. indica* can serve as a bio-inoculant to boost forage productivity and quality in *B. decumbens* by enhancing root growth and stress tolerance. The underlying mechanisms also provide a basis for developing similar bio-inoculants for other forage species, promoting more sustainable livestock production.

## 5. Conclusions

In this study, we characterized physiological, transcriptomic, and metabolomic responses of *B. decumbens* seedlings to *P. indica* colonization. *P. indica* successfully colonized *B. decumbens* roots and promoted root growth, accompanied by enhanced antioxidant enzyme activities and reduced lipid peroxidation. Colonized roots showed increased indole-3-acetic acid (IAA) levels, whereas jasmonic acid (JA) and the ethylene precursor 1-aminocyclopropane-1-carboxylic acid (ACC) were reduced. Transcriptome profiling indicated that *P. indica*-responsive genes were enriched in pathways related to secondary metabolism as well as carbohydrate- and lipid-associated processes, and multiple transcription factor families (e.g., AP2/ERF, MYB, NAC, and bZIP) were differentially expressed. Integrative transcriptome and metabolome analyses further highlighted phenylpropanoid biosynthesis and α-linolenic acid metabolism as consistently responsive pathways during colonization. Together, these results provide a multi-layered resource and a framework for mechanistic studies of how *P. indica* reshapes root physiology and metabolism in *B. decumbens*. Future studies will evaluate the role of *P. indica* in conferring stress tolerance through field trials, particularly under conditions of soil salinization and nutrient deficiency. These efforts will also focus on optimizing *P. indica*-based bio-inoculants for application across diverse forage species and environmental contexts, thereby advancing sustainable agricultural and livestock production systems.

## Data Availability

The RNA-seq raw datasets generated during the current study have been deposited in the NCBI repository under BioProject code PRJNA1372385. The datasets supporting the conclusions of this article are included within the article and its additional files.

## Supplementary Materials

The following supporting information can be downloaded at: https://www.mdpi.com/article/10.3390/biology15030215/s1, Figure S1: K-means clustering of gene expression patterns across sample groups. Expression values were Z-score-scaled (based on log-transformed expression values as described in Materials and Methods). The black line indicates the mean trend of genes within each cluster. Figure S2: qRT-PCR validation of RNA-seq results. (A) Expression patterns of ten selected unigenes determined by qRT-PCR and RNA-seq. Lines indicate qRT-PCR results, and bars indicate RNA-seq expression values (FPKMs or normalized expression as stated). (B) Correlation between log_2_ fold changes estimated from RNA-seq and qRT-PCR across the ten genes. Each point represents the mean of three biological replicates. Figure S3: GO enrichment of DEGs in *B. decumbens* roots under *P. indica* colonization. (A) Pi10d vs. CK10d; (B) Pi20d vs. CK20d; (C) Pi20d vs. Pi10d. Figure S4: Distribution of differentially expressed transcription factors (TFs) in *B. decumbens* roots under *P. indica* colonization. (A) Pi10d vs. CK10d; (B) Pi20d vs. CK20d; (C) Pi20d vs. Pi10d. Figure S5: PCA of metabolomic profiles. PCA of metabolite abundances across the four groups (CK10d, Pi10d, CK20d, and Pi20d). Figure S6: Hierarchical clustering of samples based on metabolite abundances. Clustering was performed using Z-score-normalized metabolite abundances. Color intensity represents relative metabolite levels. Figure S7: OPLS-DA of metabolomic profiles and model validation. (A–C) OPLS-DA score plots for Pi10d vs. CK10d, Pi20d vs. CK20d, and Pi20d vs. Pi10d. (D–F) Permutation tests for the corresponding OPLS-DA models. Figure S8: K-means clustering of differentially accumulated metabolites (DAMs). DAM abundances were Z-score-scaled (based on log-transformed metabolite intensities as described in Materials and Methods). The black line indicates the mean trend of metabolites within each cluster. Table S1: The qRT-PCR primers used in this study. Table S2: Alignment information for RNA-seq data. Table S3: Summary of assembly quality for RNA-Seq. Table S4: Summary of RNA-Seq data for 12 samples in this study. Table S5: Information of function annotation. Table S6: The DEGs identified in the Pi10d vs. CK10d group. Table S7: The DEGs identified in the Pi20d vs. CK20d group. Table S8: The DEGs identified in the Pi20d vs. Pi10d group. Table S9: Enriched KEGG pathways of DEGs responding to colonization by *P. indica*. Table S10: Identification of TFs in three comparison groups. Table S11: Raw data of metabolites by UPLC-MS/MS analysis. Table S12: The DAMs identified in Pi10d vs. CK10d. Table S13: The DAMs identified in Pi20d vs. CK20d. Table S14: The DAMs identified in Pi20d vs. Pi10d. Table S15: Enriched KEGG pathways of DAMs responding to colonization by *P. indica*. Table S16: The co-enriched KEGG pathway of DEGs and DAMs.

## Abbreviations

The following abbreviations are used in this manuscript:

CAT	catalase
SOD	superoxide dismutase
POD	peroxidase
MDA	malondialdehyde
IAA	indole-3-acetic acid
JA	jasmonic acid
ACC	the ethylene precursor 1-aminocyclopropane-1-carboxylic acid
DEGs	differentially expressed genes
DAMs	differentially accumulated metabolites
MAMP	microbe-associated molecular pattern
GABA	γ-aminobutyric acid
PDA	potato dextrose agar
TBA	thiobarbituric acid
NBT	nitro-blue tetrazolium
ELISAs	enzyme-linked immunosorbent assays
KEGG	Kyoto Encyclopedia of Genes and Genomes
GO	Gene Ontology
Nr	NCBI non-redundant protein
KOG	Eukaryotic Orthologous Group
FPKMs	fragments per kilobase of transcript per million mapped reads
FDR	false discovery rate
FC	genes with fold change
TFs	transcription factors
O2PLS	two-way orthogonal PLS
PCA	principal component analysis
qRT-PCR	quantitative real-time PCR
BP	biological process
MF	molecular function
CC	cellular component
CAD	cinnamyl alcohol dehydrogenase
CCR	cinnamoyl-CoA reductase
PTAL	phenylalanine/tyrosine ammonia-lyase
C3′H	p-coumarate 3-hydroxylase
COMT	caffeoyl-CoA 3-O-methyltransferase
HCT	shikimate O-hydroxycinnamoyltransferase
REF1	coniferyl-aldehyde dehydrogenase
DAD1	phospholipase A1
OPCL1	OPC-8:0 CoA ligase
LOX2S	lipoxygenase
ADH1	alcohol dehydrogenase
ACX	acyl-CoA oxidase
SPLA2	secretory phospholipase A2
MFP2	enoyl-CoA hydratase
