# Supplementary figures and images for: Physiological, Transcriptomic, and Metabolomic Responses of Brachiaria decumbens Roots During Symbiosis Establishment with Piriformospora indica
Source: Biology (Basel). 2026 Jan 23;15(3):215. doi: 10.3390/biology15030215 (PMC12896532; doi:10.3390/biology15030215)

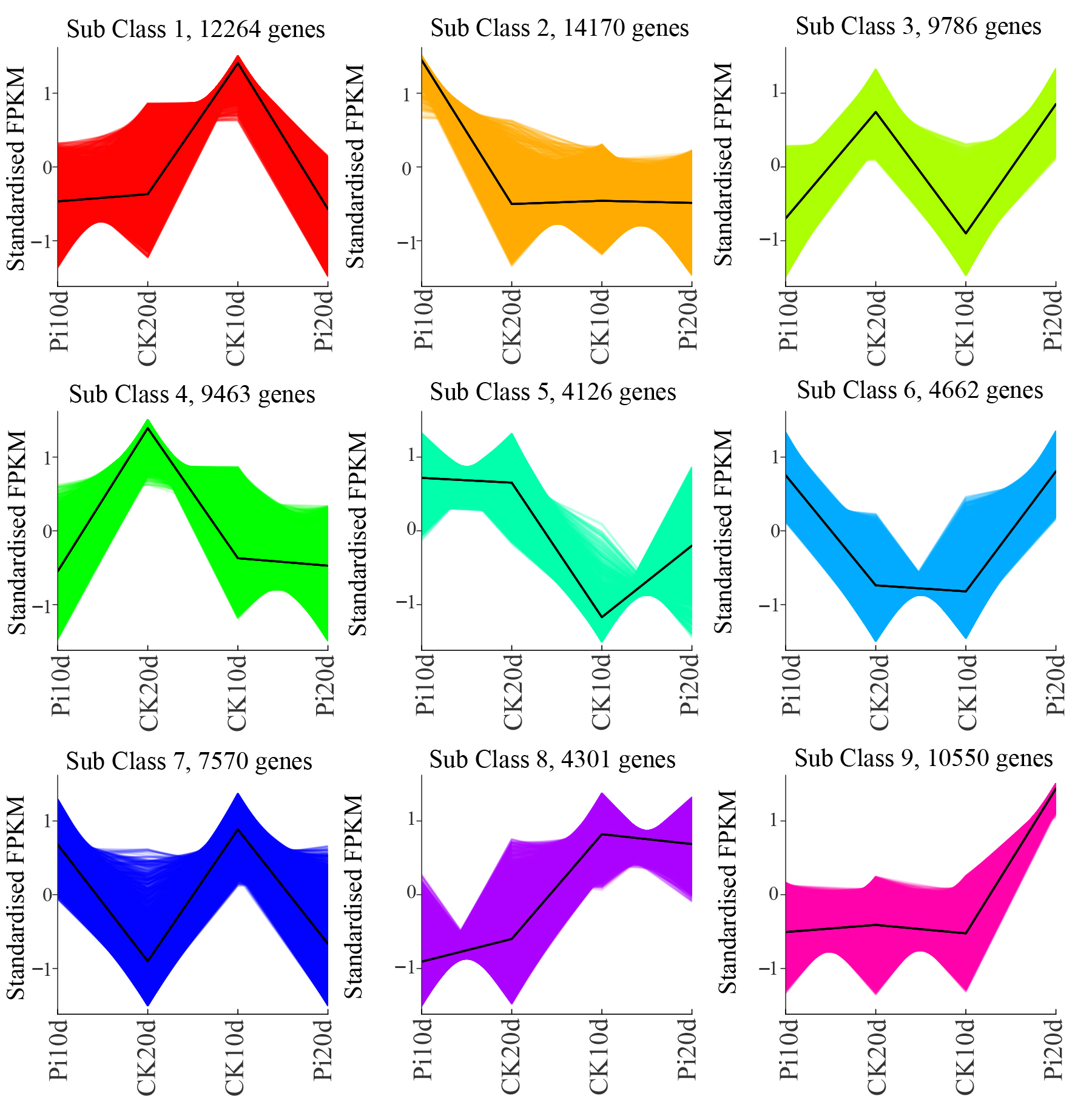

Supplement: Supplementary file 1 [file biology-15-00215-s001.zip › Figure S1.jpg]

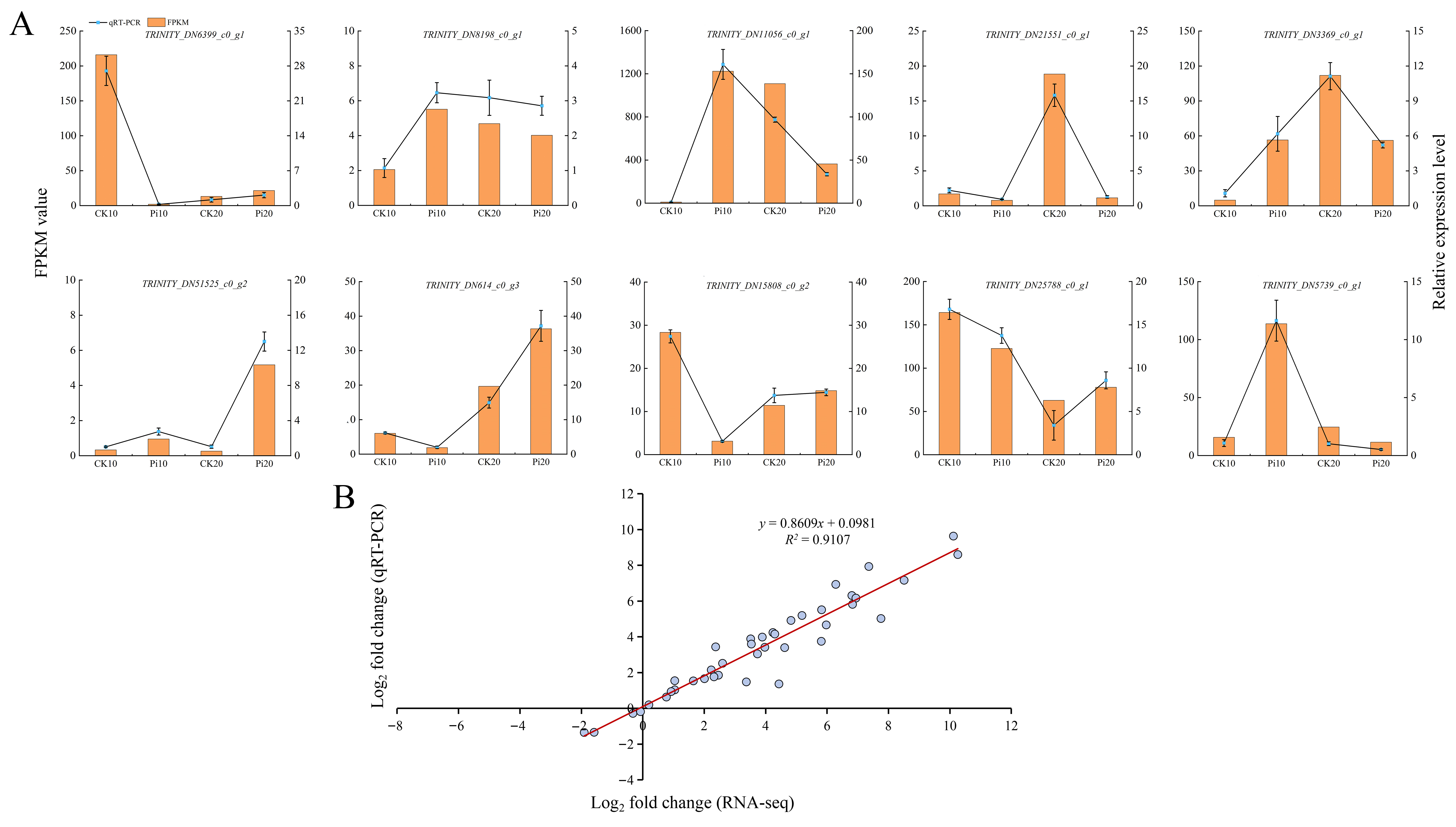

Supplement: Supplementary file 1 [file biology-15-00215-s001.zip › Figure S2.jpg]

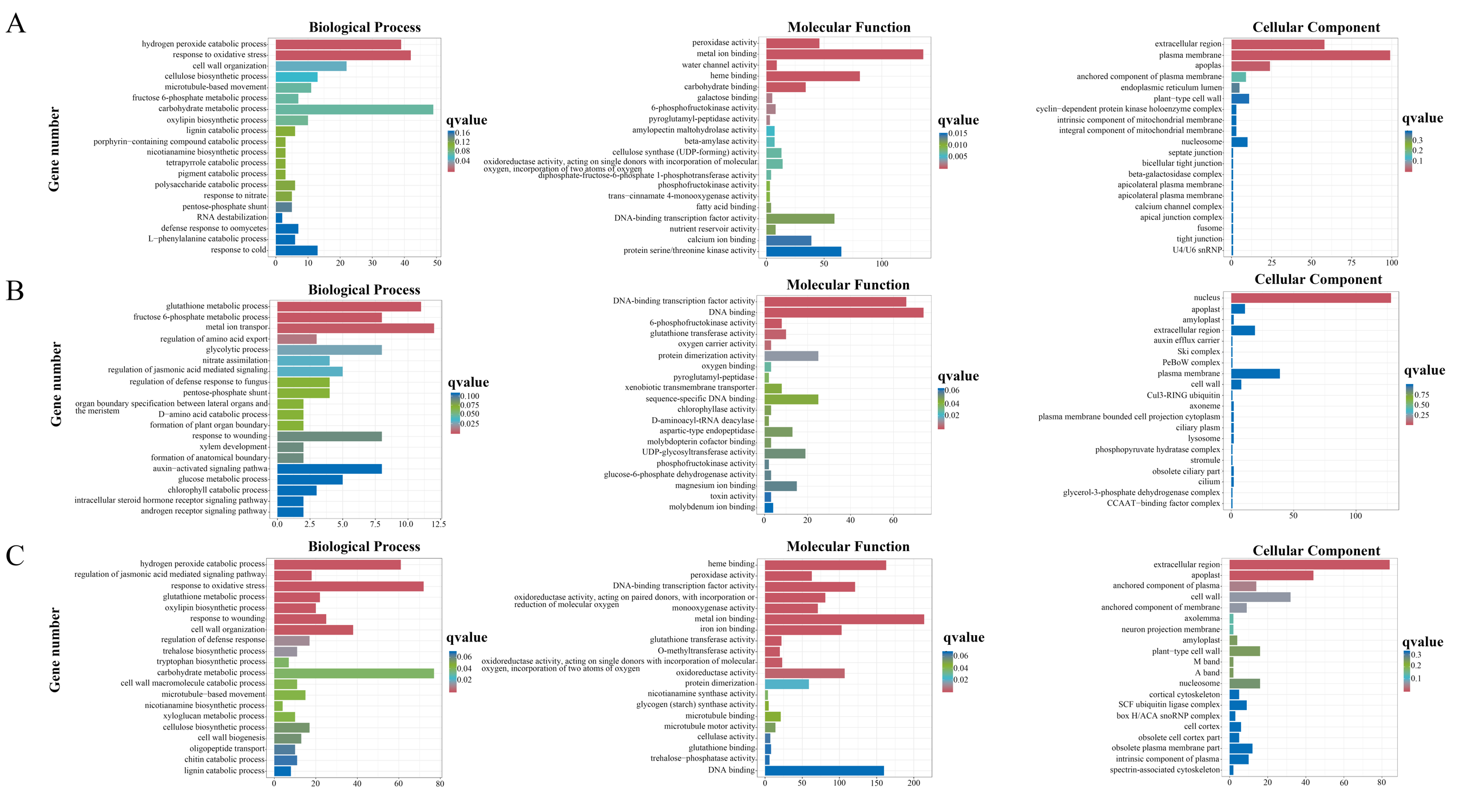

Supplement: Supplementary file 1 [file biology-15-00215-s001.zip › Figure S3.jpg]

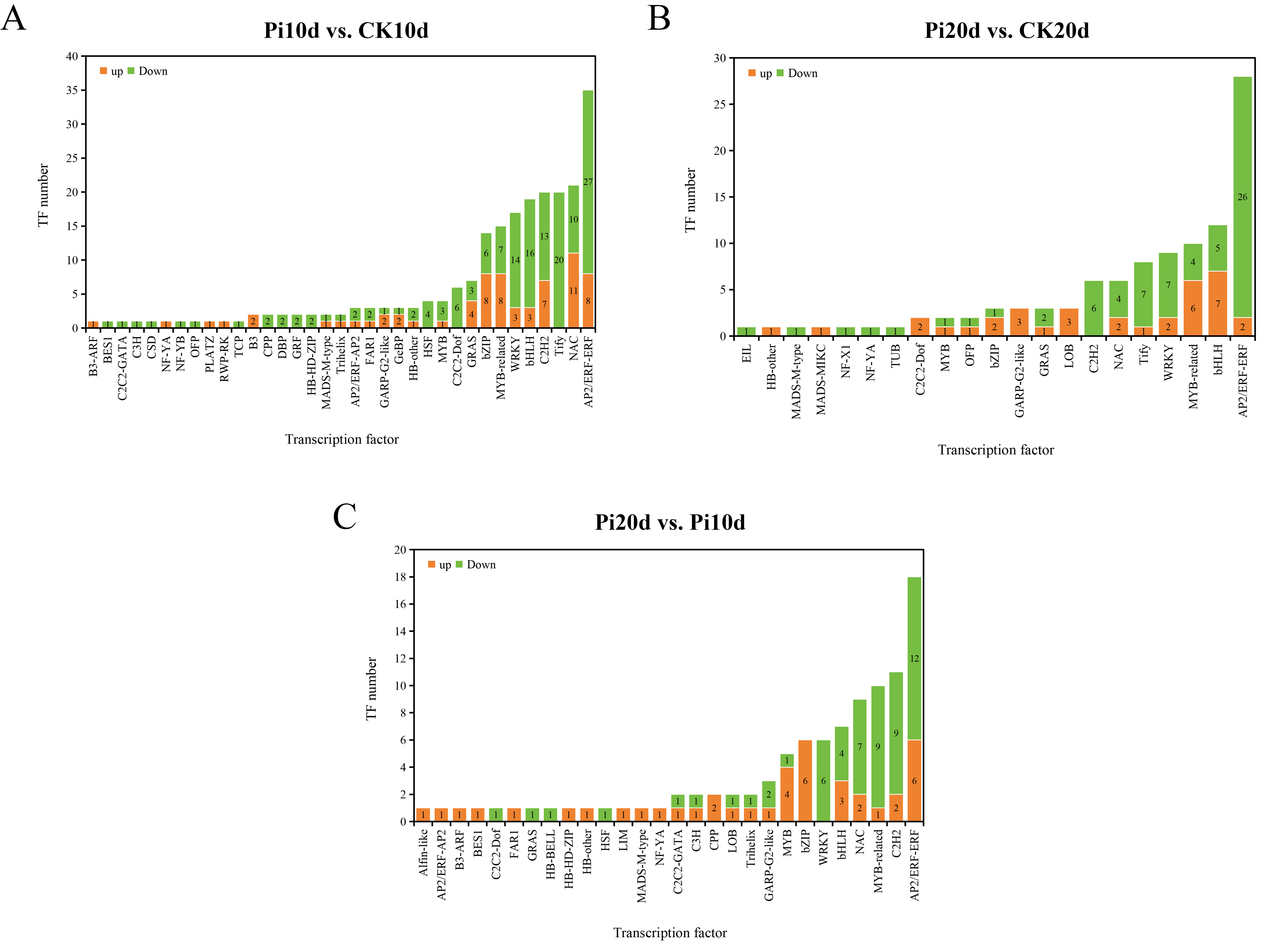

Supplement: Supplementary file 1 [file biology-15-00215-s001.zip › Figure S4.jpg]

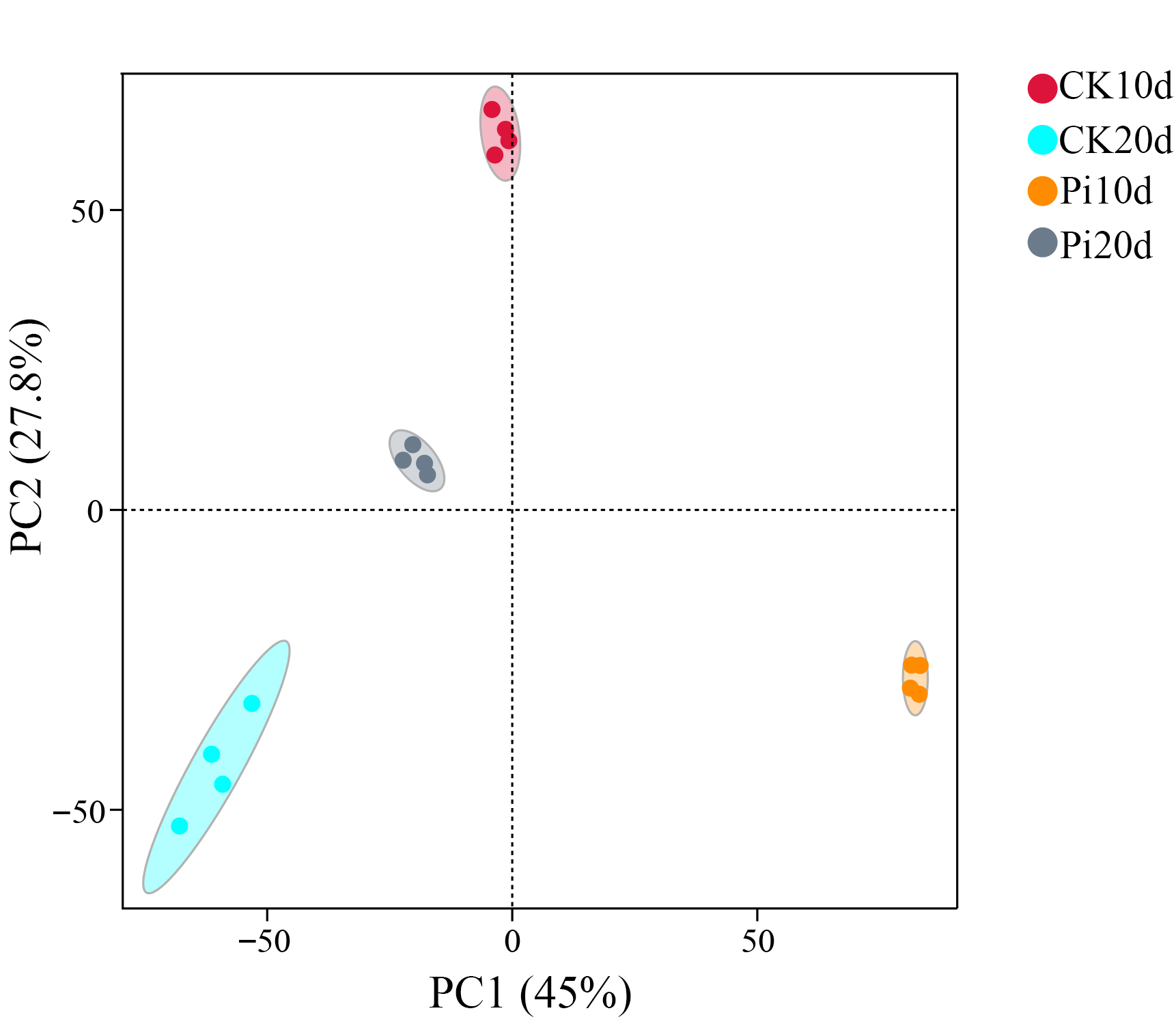

Supplement: Supplementary file 1 [file biology-15-00215-s001.zip › Figure S5.jpg]

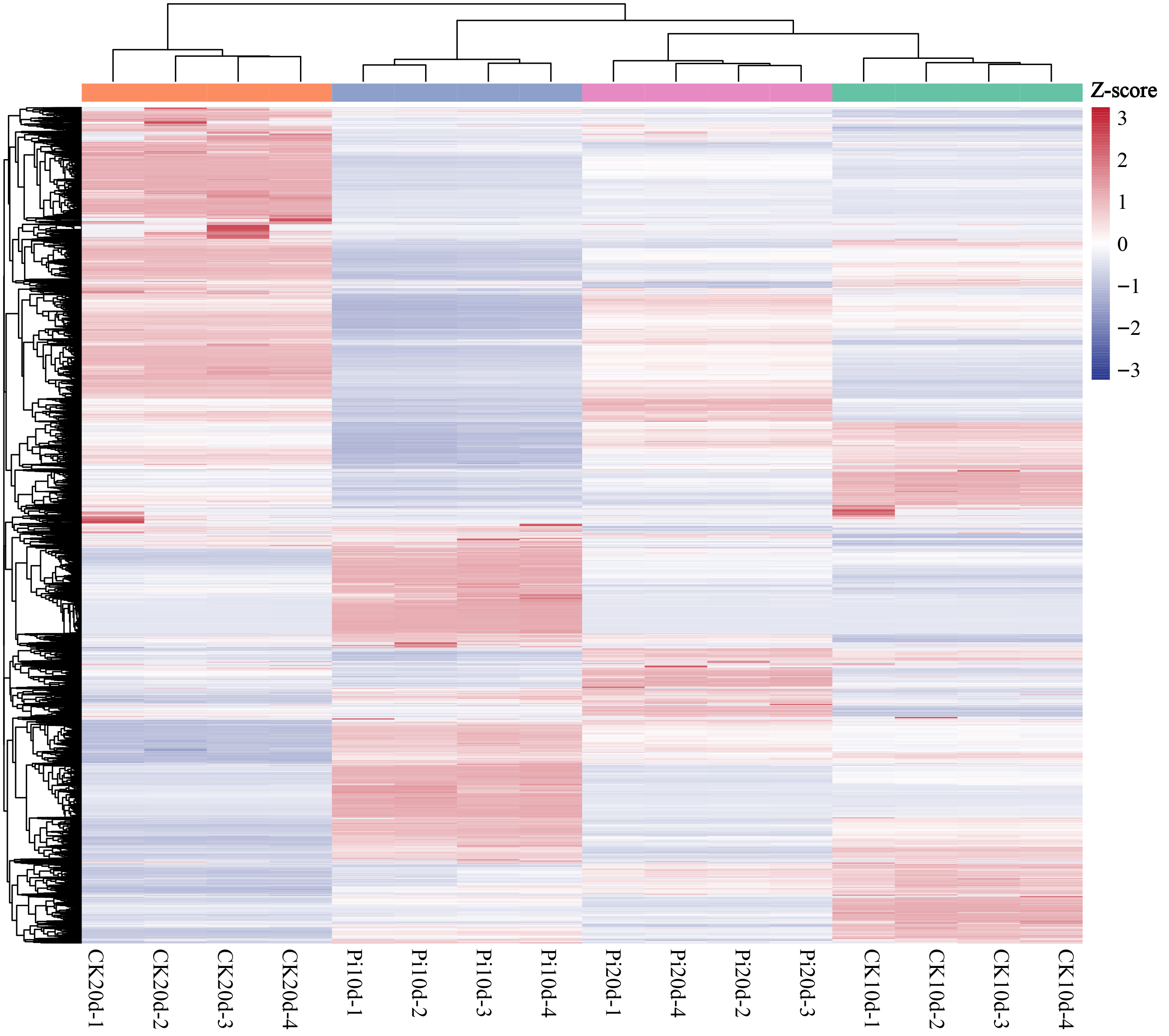

Supplement: Supplementary file 1 [file biology-15-00215-s001.zip › Figure S6.jpg]

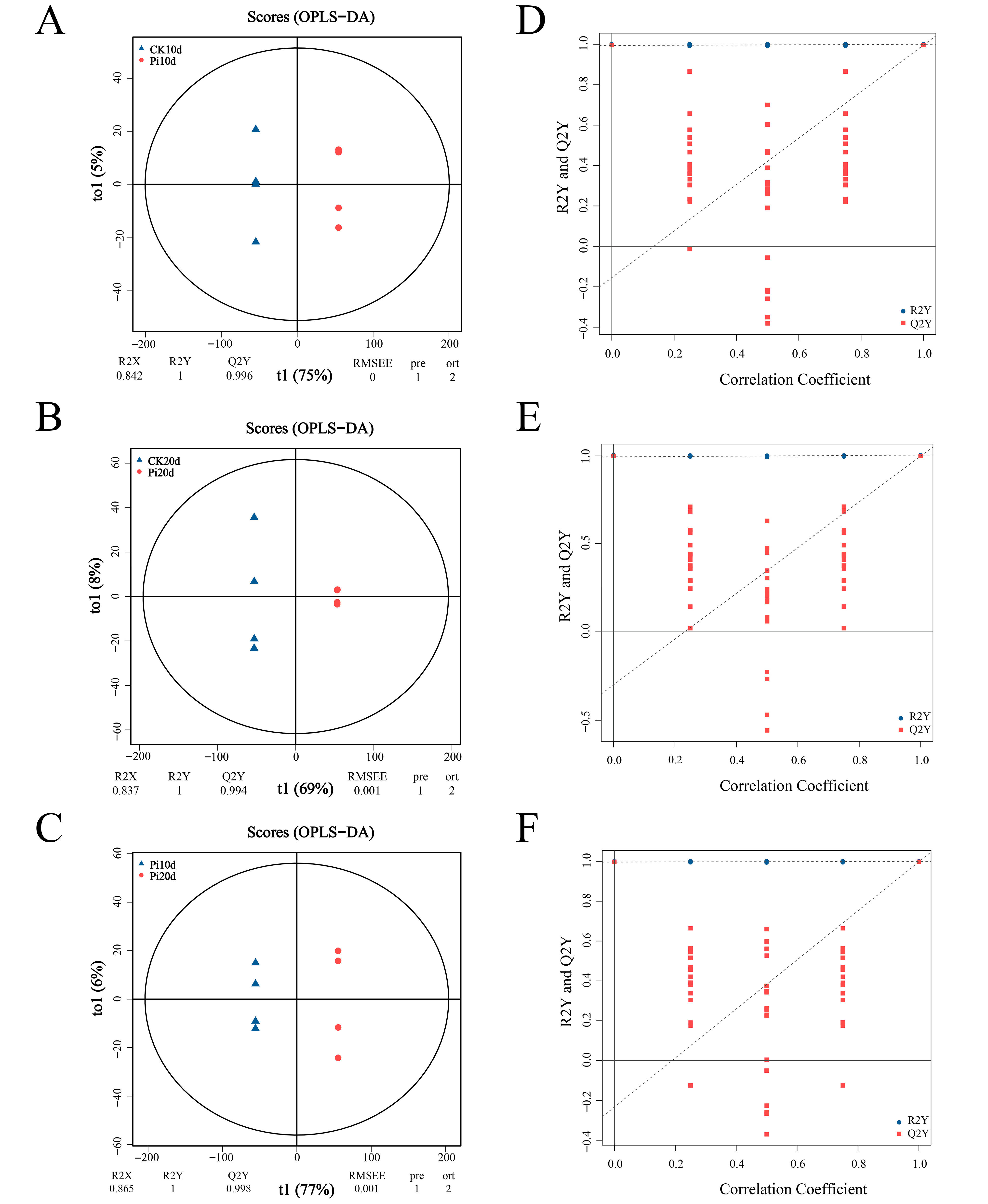

Supplement: Supplementary file 1 [file biology-15-00215-s001.zip › Figure S7.jpg]

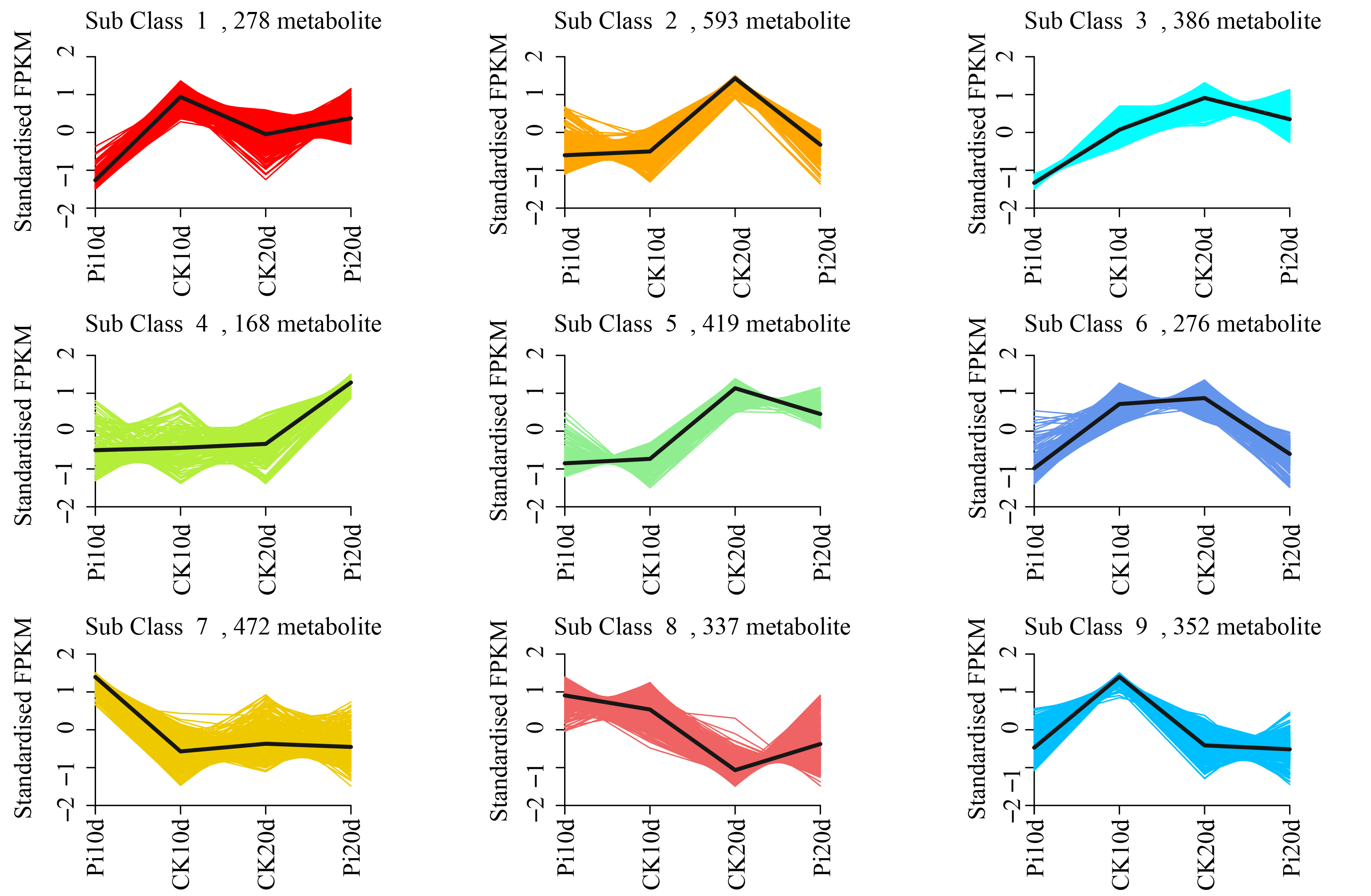

Supplement: Supplementary file 1 [file biology-15-00215-s001.zip › Figure S8.jpg]
